# PHA-665752’s Antigrowth and Proapoptotic Effects on HSC-3 Human Oral Cancer Cells

**DOI:** 10.3390/ijms25052871

**Published:** 2024-03-01

**Authors:** Anil Kumar Yadav, Saini Wang, Young-Min Shin, Byeong-Churl Jang

**Affiliations:** 1 Department of Molecular Medicine, College of Medicine, Keimyung University, 1095 Dalgubeoldaero, Dalseo-gu, Daegu 42601, Republic of Korea; yadav127@umn.edu (A.K.Y.); saini0920@naver.com (S.W.); 2 The Hormel Institute, University of Minnesota, Austin, MN 55455, USA; 3 Department of Dentistry, College of Medicine, Keimyung University, 1095 Dalgubeoldaero, Dalseo-gu, Daegu 42601, Republic of Korea

**Keywords:** PHA-665752, HSC-3, c-Met, Mcl-1, Src, HIF-1α

## Abstract

c-Met is a tyrosine-kinase receptor, and its aberrant activation plays critical roles in tumorigenesis, invasion, and metastatic spread in many human tumors. PHA-665752 (PHA) is an inhibitor of c-Met and has antitumor effects on many hematological malignancies and solid cancers. However, the activation and expression of c-Met and its role and the antitumor effect of PHA on human oral squamous cell carcinoma (OSCC) cells remain unclear. Here, we investigated the activation and expression of c-Met and the effects of PHA on the growth of a highly tumorigenic HSC-3 human OSCC cell line with high c-Met phosphorylation and expression. Of note, c-Met was highly expressed and phosphorylated on Y1234/1235 in HSC-3 cells, and PHA treatment significantly suppressed the growth and induced apoptosis of these cells. Moreover, PHA that inhibited the phosphorylation (activation) of c-Met further caused the reduced phosphorylation and expression levels of Src, protein kinase B (PKB), mammalian target of rapamycin (mTtor), and myeloid cell leukemia-1 (Mcl-1) in HSC-3 cells. In addition, the antiangiogenic property of PHA in HSC-3 cells was shown, as evidenced by the drug’s suppressive effect on the expression of hypoxia-inducible factor-1α (HIF-1α), a critical tumor angiogenic transcription factor. Importantly, genetic ablation of c-Met caused the reduced growth of HSC-3 cells and decreased Src phosphorylation and HIF-1α expression. Together, these results demonstrate that c-Met is highly activated in HSC-3 human oral cancer cells, and PHA exhibits strong antigrowth, proapoptotic, and antiangiogenic effects on these cells, which are mediated through regulation of the phosphorylation and expression of multiple targets, including c-Met, Src, PKB, mTOR, Mcl-1, and HIF-1α.

## 1. Introduction

Oral squamous cell carcinoma (OSCC) is one of the most prevalent malignant tumors of head and neck cancer, with a 5-year survival rate of only 68.5%, and is mostly found in developing countries [1,2]. Remarkably, the United States has reported 54,540 new instances cases of OSCC in 2023. Despite recent advancements in surgical and radiation therapy approaches [3,4], there has been little progress in improving the overall survival of patients with OSCC [5]. Consequently, it is important to investigate novel molecular targets to develop more potent treatment plans for patients with OSCC.

c-Met is a tyrosine-kinase receptor for hepatocyte growth factor/scatter factor (HGF/SF), and both receptor and ligand are expressed in different tissues [2,3]. The binding of HGF/SF to the extracellular domain of c-Met causes multimerization of the receptor and phosphorylation of tyrosine residues at the juxtamembrane and cytoplasmic regions, which is followed by recruitment and phosphorylation of multiple adaptor proteins, such as growth factor receptor-bound protein *2* (Grb2) and Grb-2-associated binder 1 (Gab1), as well as activation of signaling molecules, including phosphatidylinositol-3-OH kinase (PI3-K), phospholipase C-γ (PLC-γ), signal transducer and activator of transcription 3 (STAT3), extracellular signal-regulated kinase 1/2 (ERK-1/2), and focal adhesion kinase (FAK) [4,5,6,7,8]. c-Met signaling is essential for normal cell proliferation, migration, angiogenesis, embryogenesis, organogenesis, and tissue regeneration. In addition, there is now considerable evidence suggesting that aberrant c-Met signaling, resulting from mutation or overexpression of the c-Met proto-oncogene and its ligand, plays critical roles in tumorigenesis, invasion, and metastatic spread in many human tumors [9,10].

The use of c-Met inhibitors in oncology has shown promising results in preclinical and early clinical studies, including lung, breast, liver, and kidney cancers [11,12,13,14]. Previously, it has been demonstrated that c-Met is highly expressed and activated in OSCC [15], and JNJ38877605 (JNJ), a selective c-Met inhibitor, suppresses the viability and migration and promotes apoptosis in two human OSCC cell lines (HN30, CAL-27) [15]. It also has been reported that SU11274 (SU), another selective c-Met inhibitor, restrains the growth of colorectal carcinoma, which expresses a high level of c-Met [16], and it has antitumor activity in preclinical models of lung, breast, and liver cancer [17,18,19]. There is further evidence that INCB28060 (INCB), a selective inhibitor of c-Met, suppresses the growth of gastric cancer cells, which is driven by c-Met activation [14]. Moreover, studies have demonstrated that PHA-665752 (PHA), another selective and ATP-competitive c-Met inhibitor, weakens c-Met-mediated growth, motility, invasion, and morphology of ovarian, stomach, and lung cancers [9,10,20]. However, despite their potential, c-Met inhibitors have several limitations in oncology due to resistance development, limited patient selection, adverse effects, and lack of monotherapy efficacy. For example, there is limited clinical data available to assess the efficacy of SU monotherapy in oncology compared with PHA, and in a phase I trial involving patients with advanced solid tumors, PHA demonstrated acceptable safety and preliminary evidence of antitumor activity, including partial responses in lung and gastric cancer patients [21,22].

Currently, c-Met expression and phosphorylation in human OSCC cell lines and its role in their growth remain unclear. Hence, in this study, we investigated the phosphorylation and expression levels of c-Met in four different human OSCC cell lines (YD-8 and YD-38, non-tumorigenic [23]; YD-10B and HSC-3, tumorigenic [24]) and evaluated the effect of four different c-Met inhibitors mentioned above on the growth of a human OSCC cell line with highest expression and phosphorylation levels of c-Met. Here, we found that both YD-8 and HSC-3 cells have much higher phosphorylation and expression levels of c-Met than those of YD-10B and YD-38 cells and that PHA exhibits strong antisurvival, proapoptotic, and antiangiogenic effects on HSC-3 cells, mediated through regulation of the phosphorylation and expression levels of c-Met, Src, protein kinase B (PKB), mammalian target of rapamycin (mTOR), myeloid cell leukemia-1 (Mcl-1), and hypoxia-inducible factor-1α (HIF-1α).

## 2. Results

### 2.1. High Phosphorylation and Expression Levels of c-Met Are Detected in HSC-3 Human Oral Cancer Cells, and PHA Treatment at 5 μM Strongly Inhibits the Growth and Induces Apoptosis of These Cells

**Figure 1:**
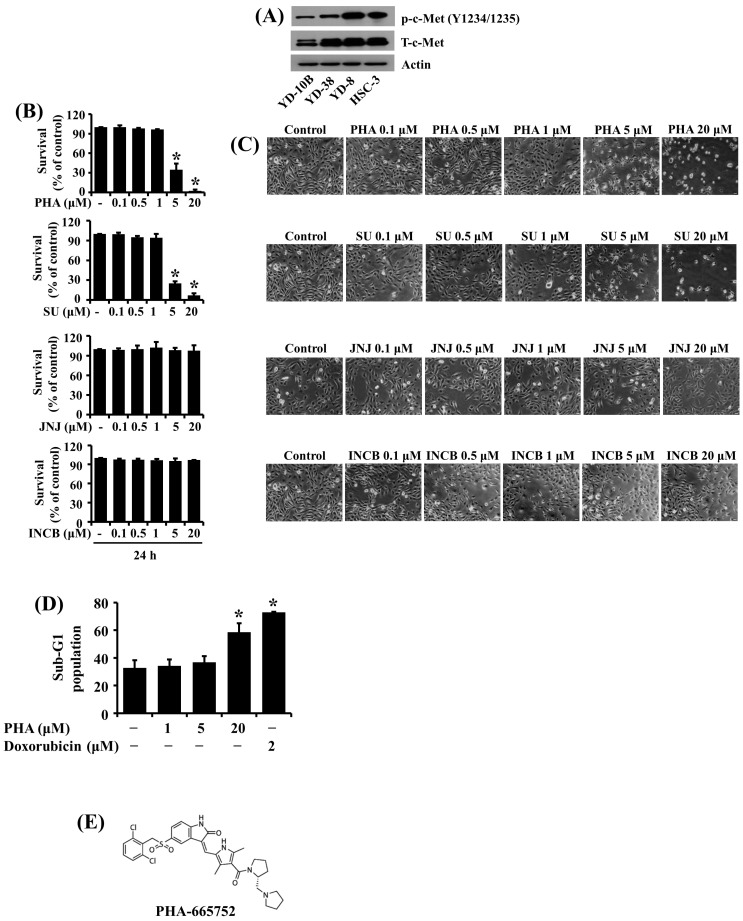
Differential phosphorylation and expression levels of c-Met in four human OSSC cell lines and abilities of PHA-665752 to inhibit the growth and induce apoptosis of HSC-3 cells. (**A**) Measurement of the phosphorylation and expression levels of c-Met in YD-10B, YD-38, YD-8, and HSC-3 cells by Western blotting. (**B**) HSC-3 cells were treated with vehicle control (DMSO; 0.1%), PHA-665752 (PHA), SU11274 (SU), JNJ38877605 (JNJ), or INCB28060 at the indicated concentrations for 24 h. The number of cells that survived was determined by cell counting assay. Experiments were performed in triplicate. Data are the means ± SE of three independent experiments. * *p* < 0.05 compared with the value of vehicle control at the indicated time. (**C**) Images of the conditioned cells in (**B**) were obtained by phase-contrast microscope. Magnification, 200× (scale bar, 50 µm). Each image is a representative of three independent experiments. (**D**) HSC-3 cells were treated with vehicle control, PHA, or doxorubicin at the indicated concentrations for 24 h. The conditioned cells were harvested and subjected to fluorescence-activated cell sorting (FACS) analysis to measure the population of the sub-G1 phase. Data are the means ± SE of three independent experiments. * *p* < 0.05 compared with the value of vehicle control at the indicated time. (**E**) The chemical structure of PHA.

We initially investigated the phosphorylation and expression levels of c-Met in four human oral cancer cell lines (YD-10B, YD-38, YD-8, and HSC-3) using Western blotting. Among the cell lines tested, YD-8 and HSC-3 cells had much higher phosphorylation and expression levels of c-Met than those of YD-10B and YD-38 cells (Figure 1A). Considering that HSC-3 cells are more aggressive and tumorigenic than YD-8 cells, we selected HSC-3 cells for further studies. We next evaluated the effect of four different c-Met inhibitors, including PHA, SU, JNJ, and INCB, on the growth of HSC-3 cells using cell count analysis. Of interest, as shown in Figure 1B, treatment with PHA and SU at 5 and 20 μM for 24 h significantly reduced the growth of HSC-3 cells, but that with JNJ or INCB at doses tested for 24 h did not influence HSC-3 cell growth. Microscopic observations further revealed the ability of PHA or SU treatment at 5 and 20 μM for 24 h to markedly inhibit the growth of HSC-3 cells (Figure 1C). Next, we studied whether PHA treatment induces the apoptosis of HSC-3 cells using flow cytometry analysis. Doxorubicin, a well-known apoptosis inducer, was used as a positive control. As shown in Figure 1D, treatment with PHA at 20 μM for 24 h resulted in a significant accumulation of sub-G1 phase of HSC-3 cells. As expected, treatment with doxorubicin at 2 μM for 24 h also caused a substantial increase in the sub-G1 population of HSC-3 cells. The chemical structure of PHA is depicted in Figure 1E. We also tested the effect of PHA at different doses on normal human gingival fibroblast (HGF) growth. Of note, PHA treatment at 5 µM for 24 h did not significantly reduce the growth of HGFs (Supplementary Figure S1). Because of the solid and selective suppressive effects on the growth of HSC-3 cells, we chose this 5 μM concentration of PHA for further work.

### 2.2. PHA Treatment at 5 μM Strongly Inhibits the Phosphorylation of c-Met and the Expression of Mcl-1 Antiapoptotic Protein in HSC-3 Human Oral Cancer Cells

**Figure 2:**
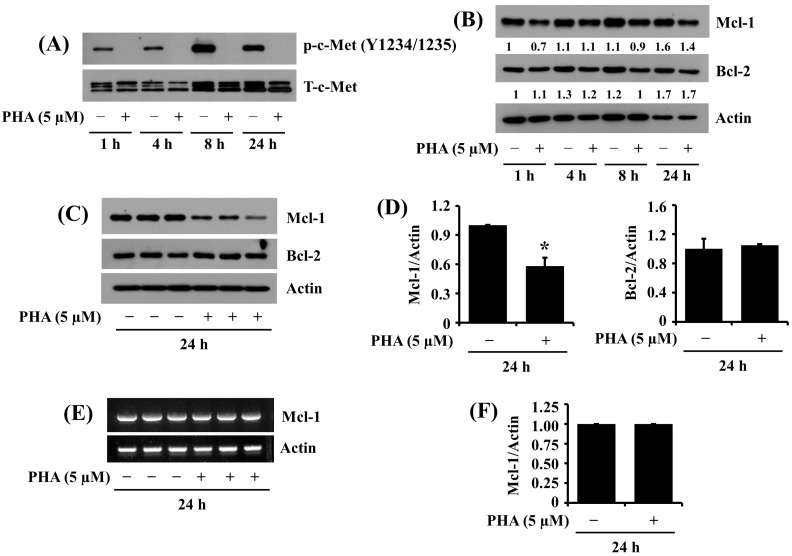
Effects of PHA on the phosphorylation and expression of c-Met, Mcl-1, and Bcl-2 in HSC-3 cells. (**A**,**B**) HSC-3 cells were treated with vehicle control (DMSO; 0.1%) or PHA (5 μM) for the indicated times. At each time point, whole-cell lysates were prepared and analyzed by Western blotting to measure the expression levels of phosphorylated (p) and total (T)-c-Met (**A**) and expression levels of Mcl-1, Bcl-2, and actin (**B**). (**C**) HSC-3 cells were treated with vehicle control or PHA (5 μM) in triplicate experiments at 24 h, followed by Western blotting to measure the protein expression levels of Mcl-1, Bcl-2, and actin. (**D**) The densitometric data in (**C**) show the protein expression levels of Mcl-1 or Bcl-2 normalized to control actin protein levels. Data are the means ± SE of three independent experiments. * *p* < 0.05 compared with vehicle control. (**E**) HSC-3 cells were treated with vehicle control or PHA (5 μM) in triplicate experiments at 24 h. Total cellular RNA was extracted and analyzed by RT-PCR to measure Mcl-1 or β-actin mRNA expression levels. (**F**) The densitometric data in (**E**) with the mRNA expression levels of Mcl-1 normalized to control actin mRNA levels. Data are the means ± SE of three independent experiments.

Given that PHA is an inhibitor of c-Met, we next examined the treatment effect of PHA at 5 μM on the phosphorylation and expression levels of c-Met in HSC-3 cells over time. Of interest, as shown in Figure 2A, in the absence of PHA, there were high phosphorylation levels of c-Met in HSC-3 cells, particularly at 8 and 24 h. However, treatment with PHA at 5 μM resulted in complete loss of the phosphorylated c-Met in HSC-3 cells at times tested, confirming the drug efficacy. Expression levels of total c-Met protein remained unchanged under these experimental conditions.

Next, to understand mechanisms associated with PHA’s antigrowth and proapoptotic effects herein, we probed whether PHA treatment affects the expression levels of Bcl-2 and Mcl-1 antiapoptotic proteins in HSC-3 cells over time. As shown in Figure 2B, in the absence of PHA, high expression levels of Bcl-2 and Mcl-1 proteins were observed in HSC-3 cells at times tested. Notably, PHA treatment at 5 μM for 8 and 24 h partially reduced Mcl-1 and Bcl-2 protein expressions in HSC-3 cells. However, results of subsequent triplicate experiments demonstrated that treatment with PHA at 5 μM for 24 h significantly reduced the expression levels of Mcl-1, but not Bcl-2, in HSC-3 cells (Figure 2C). Figure 2D is the densitometric data of expression levels of Mcl-1 or Bcl-2 protein normalized to those of actin protein in Figure 2C, obtained by Image-J software (version 1.54). We next examined whether the reduced Mcl-1 protein expression by PHA was due to a decrease in Mcl-1 transcripts in HSC-3 cells using an RT-PCR experiment. As shown in Figure 2E, the results of triplicate experiments revealed that treatment with PHA at 5 μM for 24 h did not affect Mcl-1 mRNA expression in HSC-3 cells. Figure 2F is the densitometric data of expression levels of Mcl-1 mRNA normalized to those of actin mRNA in Figure 2E, obtained by Image-J software (version 1.54).

### 2.3. PHA Treatment at 5 μM Strongly Inhibits the Phosphorylation of Src in HSC-3 Human Oral Cancer Cells

**Figure 3:**
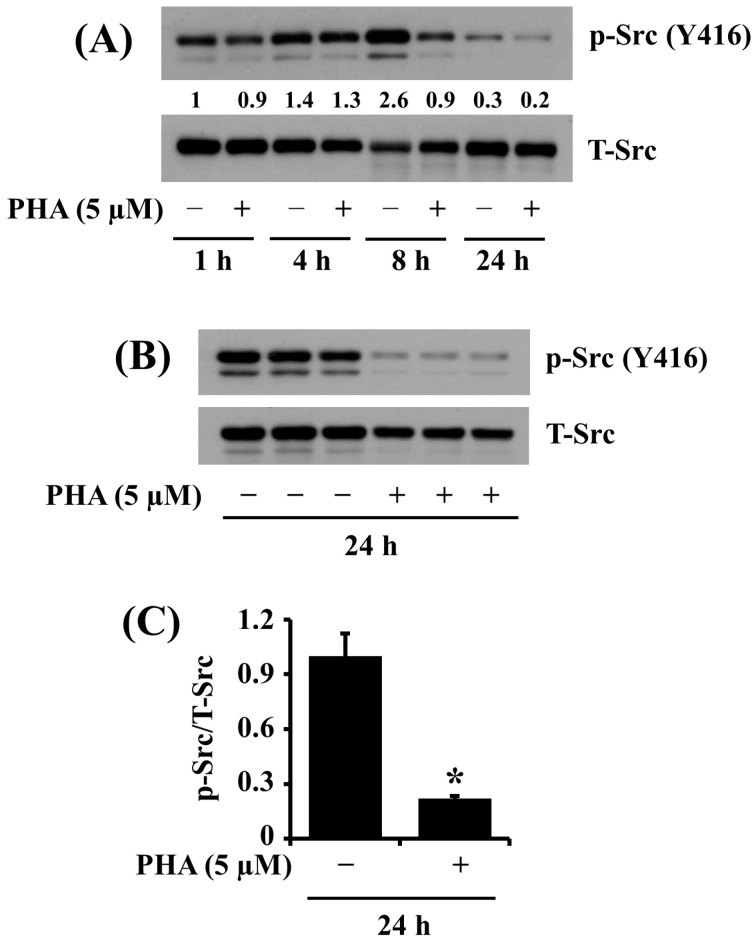
Effects of PHA on the phosphorylation and expression levels of Src in HSC-3 cells. (**A**) HSC-3 cells were treated with vehicle control (DMSO; 0.1%) or PHA (5 μM) for the indicated times. At each time point, whole-cell lysates were prepared and analyzed by Western blotting to measure the protein expression levels of phosphorylated (p) and total (T) Src. (**B**) HSC-3 cells were treated with vehicle control or PHA (5 μM) for 24 h, followed by Western blotting to measure the protein expression levels of phosphorylated (p) and total (T) Src. (**C**) The densitometric data in (**B**) with the phosphorylation levels of Src normalized to total protein expression levels of Src. Data are the means ± SE of three independent experiments. * *p* < 0.05 compared with vehicle control.

We next studied the treatment effect of PHA at 5 μM on the phosphorylation and expression level of Src, a cell survival kinase, in HSC-3 cells. Of interest, as shown in Figure 3A, in the absence of PHA, there was a time-dependent increase in phosphorylation levels of Src in HSC-3 cells at 1, 4, and 8 h and a sharp decline in protein phosphorylation at 24 h. However, PHA treatment at 5 μM led to strong loss of phosphorylated Src in HSC-3 cells at 8 and 24 h. Expression levels of the total Src protein remained constant under these experimental conditions. Image-J software (version 1.54) was used for the quantification of Western blot images. As further shown in Figure 3B, the results of triplicate experiments demonstrated the ability of PHA at 5 μM for 24 h to inhibit Src phosphorylation in HSC-3 cells significantly. Figure 3C is the densitometric data of Figure 3B with the phosphorylation levels of Src normalized to expression levels of total Src protein.

### 2.4. PHA Treatment at 5 μM Largely Downregulates the Phosphorylation and Expression of PKB and mTOR in HSC-3 Human Oral Cancer Cells

**Figure 4:**
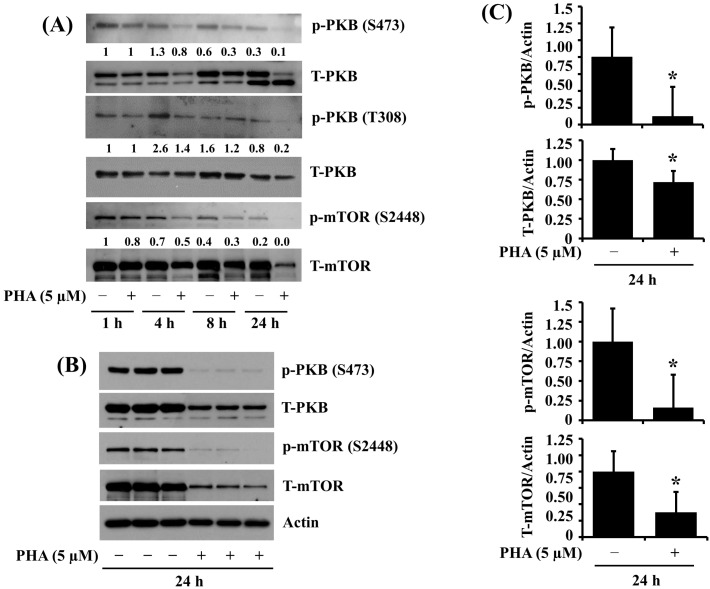
Effects of PHA on the phosphorylation and expression of PKB and mTOR in HSC-3 cells. (**A**) HSC-3 cells were treated with vehicle control (DMSO; 0.1%) or PHA (5 μM) for the indicated times. At each time point, whole-cell lysates were made and analyzed by Western blotting to measure the protein expression levels of phosphorylated (p) and total (T) PKB and mTOR. (**B**) HSC-3 cells were treated with vehicle control or PHA (5 μM) for 24 h, followed by Western blotting to measure the protein expression levels of phosphorylated (p) and total (T) PKB and mTOR, or actin. (**C**) The densitometric data in (**B**) with the phosphorylation and expression levels of PKB or mTOR normalized to protein expression levels of actin. Data are the means ± SE of three independent experiments. * *p* < 0.05 compared with vehicle control.

We further investigated the effects of PHA on the phosphorylation and expression of PKB and mTOR, cell-survival- and translation-related kinases in HSC-3 cells over time. As shown in Figure 4A, there were high phosphorylation and protein levels of PKB and mTOR in HSC-3 cells grown without PHA at the times tested. However, PHA treatment at 5 μM reduced the phosphorylation and protein expression of PKB and mTOR in HSC-3 cells. Image-J software (version 1.54) was used for the quantification of Western blot images. The triplicate experiment of Western blotting results, shown in Figure 4B, further confirmed the inhibition of phosphorylation and protein expression of PKB and mTOR by PHA in HSC cells. Figure 4C is the densitometric data of Figure 4B, with the phosphorylation and expression levels of PKB and mTOR normalized to the protein expression levels of actin, respectively.

### 2.5. PHA Downregulates HIF-1α Expression in HSC-3 Human Oral Cancer Cells

**Figure 5:**
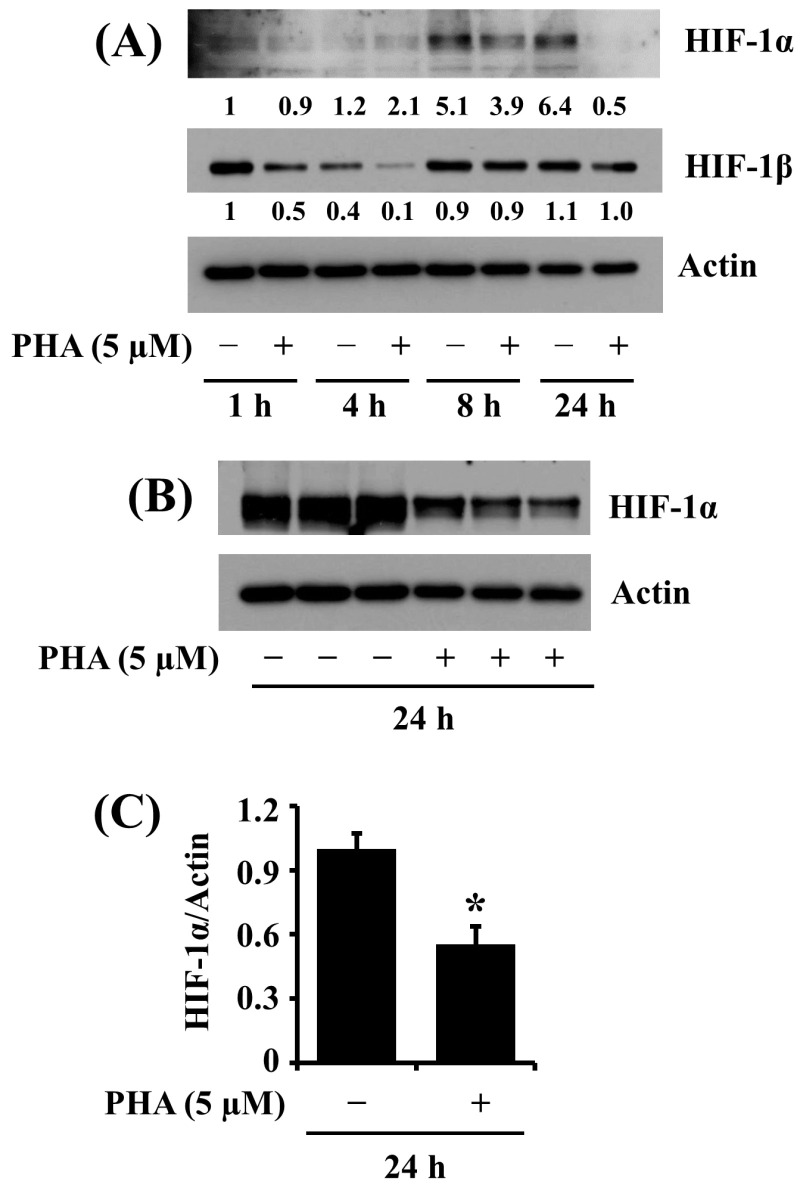
Effects of PHA on the HIF-1α expression in HSC-3 cells. (**A**,**B**) HSC-3 cells were treated with vehicle control (DMSO; 0.1%) or PHA (5 μM) for the indicated times. At each time point, whole-cell lysates were analyzed by Western blotting to measure the protein expression levels of HIF-1α and HIF-1β. (**B**) HSC-3 cells were treated with vehicle control or PHA (5 μM) in triplicate experiments at 24 h, followed by Western blotting to measure the protein expression levels of HIF-1α. (**C**) The densitometric data in (**B**) with the protein expression levels of HIF-1α normalized to protein expression levels of actin. Data are the means ± SE of three independent experiments. * *p* < 0.05 compared with vehicle control.

HIF-1α is a tumor angiogenic transcription factor [25]. Evidence suggests that HIF-1α expression is linked to tumor promotion in human OSCC [26] and correlates with the growth of human OSCC cells [27]. Previously, we have shown that the HIF-1α protein is highly expressed in HSC-3 cells under a normoxic condition [28]. This promptly led us to investigate whether PHA modulates the expression of HIF-1α in HSC-3 cells. As shown in Figure 5A, in the absence of PHA, there were high expression levels of the HIF-1α protein in HSC-3 cells at 8 and 24 h. Distinctly, PHA treatment at 5 μM resulted in almost complete downregulation of the HIF-1α protein in HSC-3 cells at 24 h. Image-J software (version 1.54) was used for the quantification of Western blot images. Data from triplicate experiments, shown in Figure 5B, further confirmed the drug’s capability to vastly lower HIF-1α protein expression in HSC cells at 24 h. Figure 5C is the densitometric data of Figure 5B, with the protein expression levels of HIF-1α normalized to those of control actin. The expression levels of the HIF-1β protein remained unchanged under these experimental conditions.

### 2.6. Knockdown of c-Met Causes the Reduced Growth of HSC-3 Human Oral Cancer Cells along with Decreased Src Phosphorylation and HIF-1α Expression

**Figure 6:**
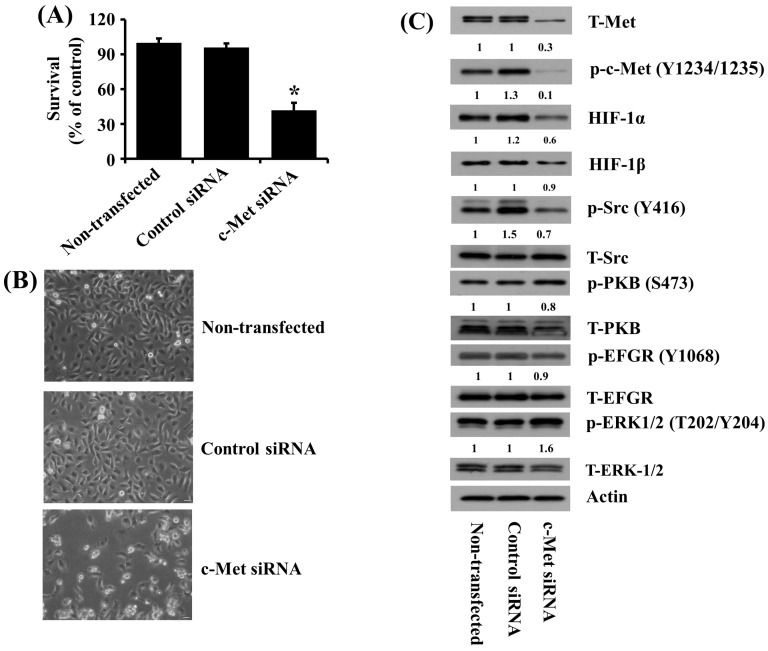
Effects of c-Met knockdown on the growth of HSC-3 cells and the expression and phosphorylation levels of c-Met, HIF-1α, HIF-1β, Src, PKB, EGFR, and ERK-1/2 in these cells. (**A**) HSC-3 cells were transfected with 100 pM of control or c-Met siRNA for 48 h, then the number of surviving cells was measured by cell count assay. The cell count assay was performed in triplicate. Data are the means ± SE of three independent experiments. * *p* < 0.05 compared with the value of vehicle control at the indicated time. (**B**) HSC-3 cells were transfected with 100 pM of control or c-Met siRNA for 48 h. Images of the conditioned cells were obtained by a phase-contrast microscope, Magnification, ×200 (scale bar, 50 µm). Each image is a representative of three independent experiments. (**C**) HSC-3 cells were transfected with 100 pM of control or c-Met siRNA for 48 h. Cells were collected, and whole-cell lysates were prepared and analyzed by Western blotting to measure the protein expression levels of phosphorylated (p) and total (T)-c-Met, Src, PKB, HIF-1α, HIF-1β, EGFR, and ERK-1/2.

To directly observe the role of c-Met expression and phosphorylation in the growth of HSC-3 cells, we transfected HSC-3 cells with control or c-Met siRNA for 48 h, followed by measurement of any change in the growth and the expression and phosphorylation levels of c-Met in control or c-Met siRNA-transfected cells. As shown in Figure 6A, the results of the cell count assay demonstrated that the knockdown of c-Met led to a significant reduction in the growth of HSC-3 cells compared with control siRNA-transfected cells. Microscopic observations also showed much fewer cells in c-Met siRNA-transfected HSC-3 cells than control siRNA-transfected cells. Moreover, Western blotting results revealed less c-Met total protein expression and phosphorylation levels in c-Met siRNA-transfected HSC-3 cells than control siRNA-transfected cells, supporting the efficiency of c-Met siRNA transfection. Notably, there were also much lower Src phosphorylation and HIF-1α expression levels in c-Met siRNA-transfected HSC-3 cells than in control siRNA-transfected cells. However, there was no or little effect on the phosphorylation and expression levels of PKB and other signaling kinases like EGFR and ERK-1/2 in c-Met siRNA-transfected HSC-3 cells compared with those in control siRNA-transfected cells. Total expression levels of HIF-1β, Src, PKB, EGFR, and ERK-1/2 remained unchanged under these experimental conditions.

## 3. Discussion

c-Met is a receptor tyrosine kinase that regulates various cellular processes, including cell survival, growth, migration, and invasion [29]. Evidence suggests that phosphorylation on tyrosine (Y) residues, such as Y1234 and Y1235, is critical for the activation of c-Met signaling [30], and abnormal c-Met activation leads to tumor development and the spread of cancer cells [31]. Several c-Met inhibitors have also been reported to have anticancer properties in solid cancer [32]. However, currently, there is minimal information regarding the phosphorylation and expression levels of c-Met and the anticancer effects and mechanisms of c-Met inhibitors in human OSCC cells. The present study demonstrated that c-Met is highly expressed and activated in tumorigenic HSC-3 human oral cancer cells. The pharmacological inhibition of c-Met by PHA or gene silencing of c-Met leads to the growth suppression and apoptosis induction of HSC-3 human oral cancer cells. Our data further indicate that PHA’s growth-suppressive, proapoptotic, and antiangiogenic effects are achieved by modulating the expression and phosphorylation of c-Met, Mcl-1, Src, PKB, mTOR, and HIF-1α.

Through initial experiments, we herein observed much higher activation and expression levels of c-Met in nontumorigenic YD-8 and tumorigenic HSC-3 cells than in tumorigenic YD-10B and nontumorigenic YD-38 cells. These results indicate differential activation and expression levels of c-Met among the four human OSCC cell lines tested. It is also worth mentioning that HSC-3 cells are highly metastatic [33]. Therefore, we selected HSC-3 cells and further explored the role of elevated activation and expression of c-Met in HSC-3 cell growth using four different c-Met inhibitors. Notably, we found that treatment with PHA or SU at 5 and 20 μM for 24 h significantly reduces the growth of HSC-3 cells, but that with JNJ or INCB at doses tested for 24 h did not influence it. These results suggest that HSC-3 cells have a differential cytotoxic response to the c-Met inhibitors tested herein.

As aforementioned, given that there are limited clinical data available to assess the efficacy of SU monotherapy in oncology compared with PHA, and PHA demonstrates acceptable safety and preliminary evidence of antitumor activity in lung and gastric cancer patients [21,22], we herein selected PHA and further investigated mechanisms by which this c-Met inhibitor modulates the growth of HSC-3 cells. Considering that inhibition of cancer cell growth is related to apoptosis [34], and PHA exerts its antitumor effect on different cancers via instigating apoptosis [33], we challenged whether PHA induces the apoptosis of HSC-3 cells. In this study, PHA leads to an increased sub-G1 phase of HSC-3 cells, which represents cells that died by apoptosis [35], supporting the drug-induced apoptosis of HSC-3 cells. Little is known about molecular targets and factors associated with PHA’s growth-suppressive and apoptosis-inducing effects on HSC-3 cells.

Mcl-1 is a Bcl-2 family of antiapoptotic proteins, crucial in regulating cell survival and apoptosis [36]. Mcl-1 is frequently overexpressed in various cancer types, and its overexpression has been implicated in promoting cancer cell survival, growth, chemoresistance, and metastasis [37]. In this study, we demonstrated that Mcl-1 and Bcl-2 proteins are substantially expressed in HSC-3 cells, and treatment with PHA significantly decreases the protein expression of Mcl-1, but not Bcl-2, in these cells, pointing out the selectivity. Of note, the results of RT-PCR in this study revealed that PHA does not affect the mRNA expression of Mcl-1 in HSC-3 cells. These data indicate that the downregulation of Mcl-1 in HSC-3 cells by PHA is due to declined protein synthesis or elevated protein turnover. Assuming that Mcl-1 acts as a cell survival and an antiapoptotic protein, it is likely that the loss of Mcl-1 may contribute to PHA’s antisurvival and proapoptotic effects herein.

Src is a nonreceptor tyrosine kinase often dysregulated in cancer cells and has been implicated in promoting cell growth, survival, and metastasis [38,39,40]. Aberrant Src activation in cancer cells is closely associated with tumor progression [39]. Of interest, it has been shown that high levels of phosphorylated Src are detected in most tongue cancer biopsies of human tongue cancer patients, and the outcome in patients with tongue cancer inversely correlates with Src hyperphosphorylation, highlighting the prognostic role of Src overexpression/hyperactivation in tongue cancer [41]. Also, in different cancers, Src signaling is reported to be a pivotal downstream transducer of c-Met-driven proliferation and growth of cells [42]. Previously, we have noted that Src is substantially expressed, and its expression is essential for the growth of HSC-3 cells [28]. In the current study, PHA treatment markedly inhibits the phosphorylation (activation) of Src in HSC-3 cells. These results confirm that Src is a survival factor in HSC-3 cells, and PHA’s antigrowth effect on HSC-3 is likely to be partially mediated through Src inhibition.

HIF-1α is a transcription factor vital in cellular response to low oxygen levels (hypoxia). In the context of cancer, HIF-1α is often upregulated in tumor cells due to the hypoxic microenvironment commonly found in solid tumors [43]. As a result, HIF-1α enables cancer cells to adapt and survive in hypoxic conditions by promoting the expression of genes involved in glycolysis, angiogenesis, cell proliferation, and survival. Under hypoxic conditions, HIF-1α stabilizes and translocates to the nucleus, where it forms a heterodimer with constitutively expressed HIF-1β and binds to hypoxia response elements in the promoter regions of target genes, including vascular endothelial growth factor [25]. It is also worth noting that HIF-1α expression is elevated in cancer cells under normoxic conditions and exposure to extracellular stimuli like growth factors [44]. Because of this, HIF-1α is currently regarded as a prime target for anticancer therapies [45]. We have previously shown that HIF-1α is highly expressed in YD-38 human oral cancer cells, and its expression is crucial for their survival [28]. In the present study, PHA significantly lowers the expression levels of HIF-1α in HSC-3 cells. Thus, it is likely that HIF-1α downregulation may further contribute to PHA’s antisurvival and antiangiogenic effects on HSC-3 cells.

PKB, also known as Akt, plays a significant role in the growth of cancer cells [46]. PKB is a serine/threonine protein kinase involved in various cellular processes, such as cell proliferation, survival, migration, and metabolism [47]. In cancer cells, aberrant activation of PKB signaling pathways, which contributes to tumor growth, progression, and resistance to therapy, has been observed [48]. PKB is often activated in cancer cells via the dysregulation of upstream signaling pathways, such as growth factor receptors like EGFR and PDGFR [49,50]. Once activated, PKB phosphorylates downstream targets, such as mTOR, that involve cell growth and survival [51]. mTOR is a serine/threonine kinase that integrates signals from growth factors, nutrients, and cellular stress to regulate various cellular processes, including increased protein synthesis and inhibition of autophagy [52]. The aberrant mTOR expression and activity in cancer cells can lead to uncontrolled cell growth and proliferation [53]. In the current study, PHA significantly reduces the phosphorylation and expression levels of PKB and mTOR in HSC-3 cells. These results show that the downregulation of PKB and mTOR may become a part of the PHA’s antisurvival effects on HSC-3 cells.

Previously, crosstalk between c-Met and other kinases or proteins, including Src, HIF-1α, PKB, and ERK-1/2, in cancer cells has been postulated [42,54,55]. In partial agreement with previous findings, the present study demonstrated that c-Met knockdown leads to decreased Src phosphorylation and HIF-1α expression, but it does not affect the phosphorylation of PKB, EGFR, and ERK-1/2 in HSC-3 cells. These results illustrate that c-Met may act as an upstream regulator of Src and HIF-1α in HSC-3 cells.

**Figure 7:**
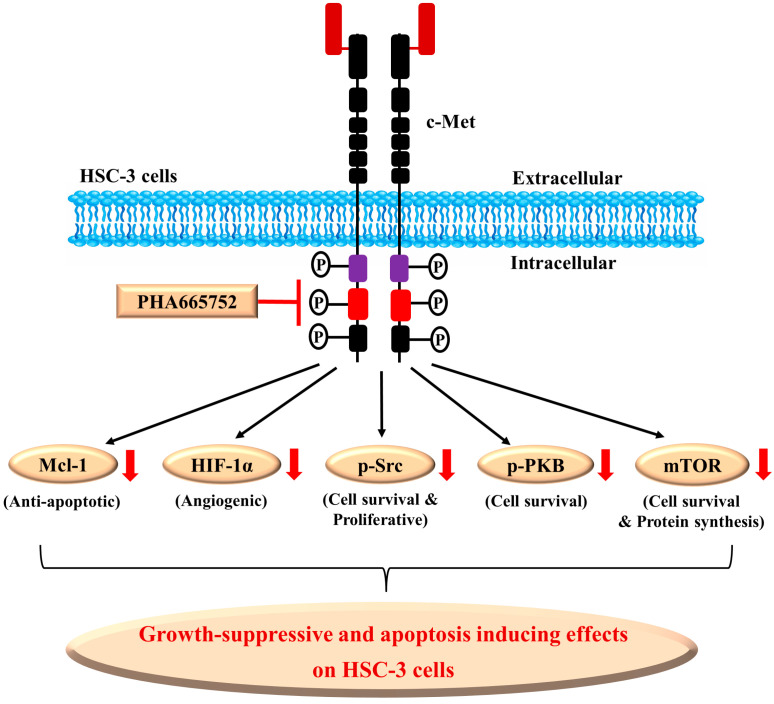
A graphical diagram of PHA-665752’s antigrowth, proapoptotic, and antiangiogenic mechanisms in HSC-3 cells. PHA may directly inhibit its target c-Met and its downstream effector pathways. PHA may also indirectly regulate additional factors, including Mcl-1, HIF-1α, Src, PKB, and mTOR. These alterations will contribute to the drug’s antisurvival, proapoptotic, and antiangiogenic effects on HSC-3 cells. The red arrows mean downregulation.

It is proposed that possible molecular and cellular mechanisms underlying the PHA’s antisurvival and proapoptotic effects on HSC-3 cells herein is that (1) PHA may directly inhibit its target c-Met and downstream effector pathways, (2) PHA may also indirectly regulate additional targets, including downregulation of an antiapoptotic protein (Mcl-1), suppression of a tumor angiogenic transcription factor (HIF-1α), and (3) PHA may decrease survival and proliferative markers (Src, PKB and mTOR), which all contribute to the drug’s antisurvival, proapoptotic, and possible antiangiogenic effects on HSC-3 cells (Figure 7).

## 4. Materials and Methods

### 4.1. Chemicals and Antibodies

Roswell Park Memorial Institute Medium (RPMI)-1640 (LM011-01), Dulbecco’s Modified Eagles Medium (DMEM; LM001-05), fetal bovine serum (FBS; S001-01), penicillin-streptomycin (LS202-02) and phosphate-buffered saline (PBS) were purchased from Welgene (Daegu, Republic of Korea). PHA-665752 (A2307), SU11274 (A2678), JNJ38877605 (943540-75-8), and INCB28060 (A8448) were purchased from ApexBio Technology (Seoul, Republic of Korea). Doxorubicin (BML-GR319) was purchased from Enzo Life Science (Seoul, Republic of Korea). Antibodies and their dilution used in this study are mentioned in Table S1 as Supplementary File. Enzyme-linked chemiluminescence (ECL) Western detection reagents were bought from Thermo Scientific (Waltham, MA, USA). Cell culture plastic wares were purchased from SPL Life Sciences (Gyeonggi-do, Republic of Korea).

### 4.2. Cell Culture

YD-38 and YD-10B cells were purchased from Korean Cell Line Bank (Seoul, Republic of Korea). HSC-3 cells were bought from ATCC (Manassas, VA, USA). Cells were grown at 37 °C in a humidified condition of 95% air and 5% CO_2_ in RPMI-1640 (YD-38 and YD-10B) or DMEM (HSC-3) media supplemented with 10% heat-inactivated FBS, 100 U/mL penicillin, and 100 μg/mL streptomycin.

### 4.3. Cell Viability and Survival Assay

For cell viability assay, cells (1 × 10^5^ cells/mL) were seeded and treated with different doses of PHA (0, 0.1, 0.5, 1, 5, and 20 μM) for 24 h. For cell survival assay, the surviving cells were counted by the trypan blue exclusion method in triplicates and expressed as percentage against control group.

### 4.4. Determination of Sub-G1 (Apoptotic) Phase

After 24 h of exposure with mock (DMSO; 0.1%), PHA (1, 5, and 20 µM) HSC-3 cells were collected and fixed in ice-cold 70% ethanol for 2 h at 4 °C. Later, cells were rinsed with PBS and stained with propidium iodide solution containing 100 μg/mL RNase A, 50 μg/mL propidium iodide and placed in dark for 30 min. Flow cytometric analyses (FACS Caliber, Becton Dickinson, Franklin Lakes, NJ, USA) were performed, and CellQuest software (version 5.2, Becton Dickinson, Franklin Lakes, NJ, USA) was used for analysis.

### 4.5. siRNA Transfection

Small interfering RNA (siRNA) of c-Met (sc-29228) and control siRNA-A (sc-37007) were obtained from Santa Cruz. HSC-3 cells were transfected with 100 pM of control or c-Met siRNA using Lipofectamine RNAiMAX (Invitrogen, Grand Island, NY, USA) according to the manufacturer’s recommendations. 48 h later, the cells were lysed, and protein expressions were detected by Western blotting.

### 4.6. Western Blot Analysis

Proteins (50 μg) were separated by SDS-PAGE and transferred onto nitrocellulose membranes (Millipore, Bedford, MA, USA). The membranes were washed with TBS (10 mM Tris-Cl, 150 mM NaCl, pH 7.5) supplemented with 0.05% (*v*/*v*) Tween 20 followed by blocking with TBST containing 5% (*w*/*v*) nonfat dried milk. The membranes were incubated overnight with antibody-specific antibodies at 4 °C. The membranes were then exposed to secondary antibodies conjugated to horseradish peroxidase (HRP) for 2 h at room temperature and further washed three times with TBST. Immunoreactivity was detected by ECL reagents (Advansta, CA, USA). Equal protein loading was assessed by the expression level of β-actin. Image-J software (version 1.54) was used for quantification of Western blot images.

### 4.7. Reverse-Transcription Polymerase Chain Reaction (RT-PCR) Analysis

Total transcript from conditioned HSC-3 cells was isolated using TRIzol^®^ (Thermo Fisher Scientific, Inc., Waltham, MA, USA) according to the manufacturer’s protocol. RT-PCR was essentially as previously described [10,21] using the respective Mcl-1 and actin primer pairs: Mcl-1 sense, 5′-ATCTCTCGGTACCT TCGGGAG-3′ and Mcl-1 anti-sense, 5′-ACCAGCTCCTACTCCAGCAAC-3′; and actin sense, 5′-TCAAGATCATTGCTCCTCCTG-3′ and actin anti-sense, 5′-CTGCTTGCTGATCCACATCTG-3′. The PCR conditions applied were the following: Mcl-1, 25 cycles of denaturation at 95 °C for 45 s, annealing at 56 °C for 45 s, and extension at 72 °C for 45 s; β-actin, 25 cycles of denaturation at 95 °C for 30 s, annealing at 56 °C for 30 s, and extension at 72 °C for 30 s. Actin was used as an internal control to evaluate the relative expression of Mcl-1.

### 4.8. Statistical Analysis

The data were expressed as means ± standard error (SE). The significance of differences was determined by one-way ANOVA using SPSS 11.5 software (SPSS, Inc., Chicago, IL, USA). All significance testing was based upon a *p* < 0.05.

## 5. Conclusions

These results demonstrate that PHA has strong antisurvival, proapoptotic, and antiangiogenic effects on HSC-3 human oral cancer cells, and these effects are mediated through control of the phosphorylation and expression of c-Met, Src, PKB, mTOR, Mcl-1, and HIF-1α. Although critical issues need to be resolved, including the antitumor effects of PHA on animal models, our present findings suggest that PHA may be used as a potential therapeutic agent for treating human OSCC with aberrant c-Met expression and activation.

## Data Availability

Data are contained within the article.

## Supplementary Materials

The following supporting information can be downloaded at https://www.mdpi.com/article/10.3390/ijms25052871/s1, Figure S1: Effects of PHA on the growth of HGFs; Table S1: List of antibodies used for Western blot analysis.

## References

[1] Sagheer S.H., Whitaker-Menezes D., Han J.Y.S., Curry J.M., Martinez-Outschoorn M., Philp N.J. (2021). 4NQO induced carcinogenesis: A mouse model for oral squamous cell carcinoma. Methods Cell Biol..

[2] Kademani D. (2021). Oral cancer. Mayo Clin. Proc..

[3] Jemal A., Siegel R., Ward E., Hao Y., Xu J., Murray T., Thun M.J. (2008). Cancer statistics, 2008. CA Cancer J. Clin..

[4] Lothaire P., de Azambuja E., Dequanter D., Lalami Y., Sotiriou C., Andry G., Castro G.J., Awada A. (2006). Molecular markers of head and neck squamous cell carcinoma: Promising signs in need of prospective evaluation. Head Neck.

[5] Ferreira A.K.A., de Carvalho S.H.G., Granville-Garcia A.F., Sarmento D.J.D.S., Agripino G.G., de Abreu M.H.N.G., de Melo M.C.F., Caldas A.D.F., Godoy G.P. (2021). Survival and prognostic factors in patients with oral squamous cell carcinoma. Med. Oral Patol. Oral Cir. Bucal.

[6] Szturz P., Raymond E., Abitbol C., Albert S., de Gramont A., Faivre S. (2017). Understanding c-MET signalling in squamous cell carcinoma of the head & neck. Crit. Rev. Oncol. Hematol..

[7] Kim B., Jung N., Lee S., Sohng J.K., Jung H.J. (2016). Apigenin inhibits cancer stem cell-like phenotypes in human Glioblastoma cells via suppression of c-Met Signaling. Phytother. Res..

[8] International Cancer Genome Consortium PedBrain Tumor Project (2016). Recurrent MET fusion genes represent a drug target in pediatric glioblastoma. Nat. Med..

[9] Hassan W., Chitcholtan K., Sykes P., Garrill A. (2016). A Combination of Two Receptor Tyrosine Kinase Inhibitors, Canertinib and PHA665752 compromises ovarian cancer cell growth in 3D cell models. Oncol. Ther..

[10] Magali H., Michaela M., Daniel M.A., Andree B., Friedhelm B., Martin F.F., Yitzhak Z., Mario P.T. (2013). Protective autophagy is involved in resistance towards MET inhibitors in human gastric adenocarcinoma cells. Biochem. Biophys. Res. Comm..

[11] Hardy-Werbin M., del Rey-Vergara R., Galindo-Campos M.A., Moliner L., Arriola E. (2019). MET inhibitors in small cell lung cancer: From the bench to the bedside. Cancers.

[12] Lefebvre C., Allan A.L. (2021). Anti-proliferative and anti-migratory effects of EGFR and c-Met tyrosine kinase inhibitors in triple negative breast cancer cells. Precis. Cancer Med..

[13] Xie Z., Lee Y.H., Boeke M., Jilaveanu L.B., Liu Z., Bottaro D.P., Kluger H.M., Shuch B. (2016). MET inhibition in clear cell renal cell carcinoma. J. Cancer.

[14] Liu X., Wang G., Yang G., Marando C., Koblish H.K., Hall L.M., Fridman J.S., Behshad E., Wynn R., Li Y. (2011). A novel kinase inhibitor, INCB28060, blocks c-MET-dependent signaling, neoplastic activities, and cross-talk with EGFR and HER-3. Clin. Cancer Res..

[15] Sun Z., Liu Q., Ye D., Ye K., Yang Z., Li D. (2018). Role of c-Met in the progression of human oral squamous cell carcinoma and its potential as a therapeutic target. Oncol. Rep..

[16] Gao W., Bing X., Han J.Y.S., Li M., Yang Z., Li Y., Chen H. (2013). Study of critical role of c-Met and its inhibitor SU11274 in colorectal carcinoma. Med. Oncol..

[17] Ma P.C., Jagadeeswaran R., Jagadeesh S., Tretiakova M.S., Nallasura V., Fox E.A., Hansen M., Schaefer E., Naoki K., Lader A. (2005). Functional expression and mutations of c-Met and its therapeutic inhibition with SU11274 and small interfering RNA in non-small cell lung cancer. Cancer Res..

[18] Horm T.M., Bitler B.G., Broka D.M., Louderbough J.M., Schroeder J.A. (2012). MUC1 drives c-Met-dependent migration and scattering. Mol. Cancer Res..

[19] Inagaki Y., Qi F., Gao J., Qu X., Hasegawa K., Sugawara Y., Tang W., Kokudo N. (2011). Effect of c-Met inhibitor SU11274 on hepatocellular carcinoma cell growth. Biosci. Trends.

[20] Yang Y., Wislez M., Fujimoto N., Prudkin L., Izzo J.G., Uno F., Ji L., Hanna A.E., Langley R.R., Liu D. (2008). A selective small molecule inhibitor of c-Met, PHA-665752, reverses lung premalignancy induced by mutant K-ras. Mol. Cancer Ther..

[21] Matsubara D., Ishikawa S., Oguni S., Aburatani H., Fukayama M., Niki T. (2010). Molecular predictors of sensitivity to the MET inhibitor PHA665752 in lung carcinoma cells. J. Thorac. Oncol..

[22] Christensen J.G., Schreck R., Burrows J., Kuruganti P., Chan E., Le P., Chen J., Wang X., Ruslim L., Blake R. (2003). A selective small molecule inhibitor of c-Met kinase inhibits c-Met-dependent phenotypes in vitro and exhibits cytoreductive antitumor activity in vivo. Cancer Res..

[23] Lee E., Kim J., Lee S., Kim E.J., Chun Y.C., Ryu M.H., Yook J.I. (2005). Characterization of newly established oral cancer cell lines derived from six squamous cell carcinoma and two mucoepidermoid carcinoma cells. Exp. Mol. Med..

[24] Erdem N.F., Carlson E.R., Gerard D.A., Ichiki A.T. (2007). Characterization of 3 oral squamous cell carcinoma cell lines with different invasion and/or metastatic potentials. J. Oral Maxillofac. Surg..

[25] Shi Y.H., Fang W.G. (2004). Hypoxia-inducible factor-1 in tumour angiogenesis. World J. Gastroenterol..

[26] Ryu M.H., Park H.M., Chung J., Lee C.H., Park H.R. (2010). Hypoxia-inducible factor-1α mediates oral squamous cell carcinoma invasion via upregulation of α5 integrin and fibronectin. Biochem. Biophys. Res. Comm..

[27] Sumera S., Ali A., Yousafzai Y.M., Durrani Z., Alorini M., Aleem B., Zahir R. (2023). Overexpression of hypoxia-inducible factor-1α and its relation with aggressiveness and grade of oral squamous cell carcinoma. Diagnostics.

[28] Park N.S., Park Y.K., Yadav A.K., Shin Y.M., Bishop-Bailey D., Choi J.S., Park J.W., Jang B.C. (2021). Anti-growth and pro-apoptotic effects of dasatinib on human oral cancer cells through multi-targeted mechanisms. J. Cell. Mol. Med..

[29] Graveel C.R., Tolbert D., Vande Woude G.F. (2013). MET: A critical player in tumorigenesis and therapeutic target. Cold Spring Harb. Perspect. Biol..

[30] Longati P., Bardelli A., Ponzetto C., Naldini L., Comoglio P.M. (1994). Tyrosines1234–1235 are critical for activation of the tyrosine kinase encoded by the MET proto-oncogene (HGF receptor). Oncogene.

[31] Zhang Y., Xia M., Jin K., Wang S., Wei H., Fan C., Wu Y., Li X., Li X., Li G. (2018). Function of the c-Met receptor tyrosine kinase in carcinogenesis and associated therapeutic opportunities. Mol. Cancer.

[32] De Bono J.S., Yap T.A. (2011). c-MET: An exciting new target for anticancer therapy. Ther. Adv. Med. Oncol..

[33] Matsui T., Ota T., Ueda Y., Tanino M., Odashima S. (1998). Isolation of a highly metastatic cell line to lymph node in human oral squamous cell carcinoma by orthotopic implantation in nude mice. Oral Oncol..

[34] Scott W., Lowe A., Lin W. (2000). Apoptosis in cancer. Carcinogenesis.

[35] Plesca D., Mazumder S., Almasan A. (2008). DNA damage response and apoptosis. Methods Enzymol..

[36] Kozopas K.M., Yang T., Buchan H.L., Zhou P., Craig R.W. (1993). MCL1, a gene expressed in programmed myeloid cell differentiation, has sequence similarity to BCL2. Proc. Natl. Acad. Sci. USA.

[37] Wang H., Guo M., Wei H., Chen Y. (2021). Targeting MCL-1 in cancer: Current status and perspectives. J. Hematol. Oncol..

[38] Wheeler D.L., Iida M., Dunn E.F. (2009). The role of Src in solid tumors. Oncologist.

[39] Summy J.M., Gallick G.E. (2003). Src family kinases in tumor progression and metastasis. Cancer Metastasis Rev..

[40] Biscardi J.S., Tice D.A., Parsons S.J. (1999). c-Src, receptor tyrosine kinases, and human cancer. Adv. Cancer Res..

[41] Ben-Izhak O., Cohen-Kaplan V., Nagler R.M. (2010). The prognostic role of phospho-Src family kinase analysis in tongue cancer. J. Cancer Res. Clin. Oncol..

[42] Zhang S., Yu D. (2012). Targeting Src family kinases in anti-cancer therapies: Turning promise into triumph. Trends Pharmacol. Sci..

[43] Semenza G. (2003). Targeting HIF-1 for cancer therapy. Nat. Rev. Cancer.

[44] Bos R., van Diest P.J., de Jong J.S., van der Groep P., van der Valk P., van der Wall E. (2005). Hypoxia-inducible factor-1alpha is associated with angiogenesis, and expression of bFGF, PDGF-BB, and EGFR in invasive breast cancer. Histopathology.

[45] Bui B.P., Nguyen P.L., Lee K., Cho J. (2022). Hypoxia-Inducible Factor-1: A novel therapeutic target for the management of cancer, drug resistance, and cancer-related pain. Cancers.

[46] Manning B.D., Cantley L.C. (2007). AKT/PKB signaling navigating downstream. Cell.

[47] Sen P., Mukherjee S., Ray D., Raha S. (2003). Involvement of the Akt/PKB signaling pathway with disease processes. Mol. Cell Biochem..

[48] Chin Y.R., Toker A. (2009). Function of Akt/PKB signaling to cell motility, invasion and the tumor stroma in cancer. Cell Signal.

[49] Nishimura Y., Takiguchi S., Ito S., Itoh K. (2015). EGF-stimulated AKT activation is mediated by EGFR recycling via an early endocytic pathway in a gefitinib-resistant human lung cancer cell line. Int. J. Oncol..

[50] Zhang H., Bajraszewski N., Wu E., Wang H., Moseman A.P., Dabora S.L., Griffin J.D., Kwiatkowski D.J. (2007). PDGFRs are critical for PI3K/Akt activation and negatively regulated by mTOR. J. Clin. Investig..

[51] Gunn R.M., Hailes H.C. (2008). Insights into the PI3-K-PKB-mTOR signalling pathway from small molecules. J. Chem. Biol..

[52] Kim Y.C., Guan K.L. (2015). mTOR: A pharmacologic target for autophagy regulation. J. Clin. Investig..

[53] Cargnello M., Tcherkezian J., Roux P.P. (2015). The expanding role of mTOR in cancer cell growth and proliferation. Mutagenesis.

[54] Organ S.L., Tsao M.S. (2011). An overview of the c-MET signaling pathway. Ther. Adv. Med. Oncol..

[55] Whang Y.M., Jung S.P., Kim M.-K., Chang I.H., Park S.I. (2019). targeting the hepatocyte growth factor and c-Met signaling axis in bone metastases. Int. J. Mol. Sci..

