# Supplementary material for: PHA-665752’s Antigrowth and Proapoptotic Effects on HSC-3 Human Oral Cancer Cells
Source: Int J Mol Sci. 2024 Mar 1;25(5):2871. doi: 10.3390/ijms25052871 (PMC10932316; doi:10.3390/ijms25052871)
Supplement: Supplementary file 1 [file ijms-25-02871-s001.zip › ijms-2828335 supplementary figure S1.pdf]

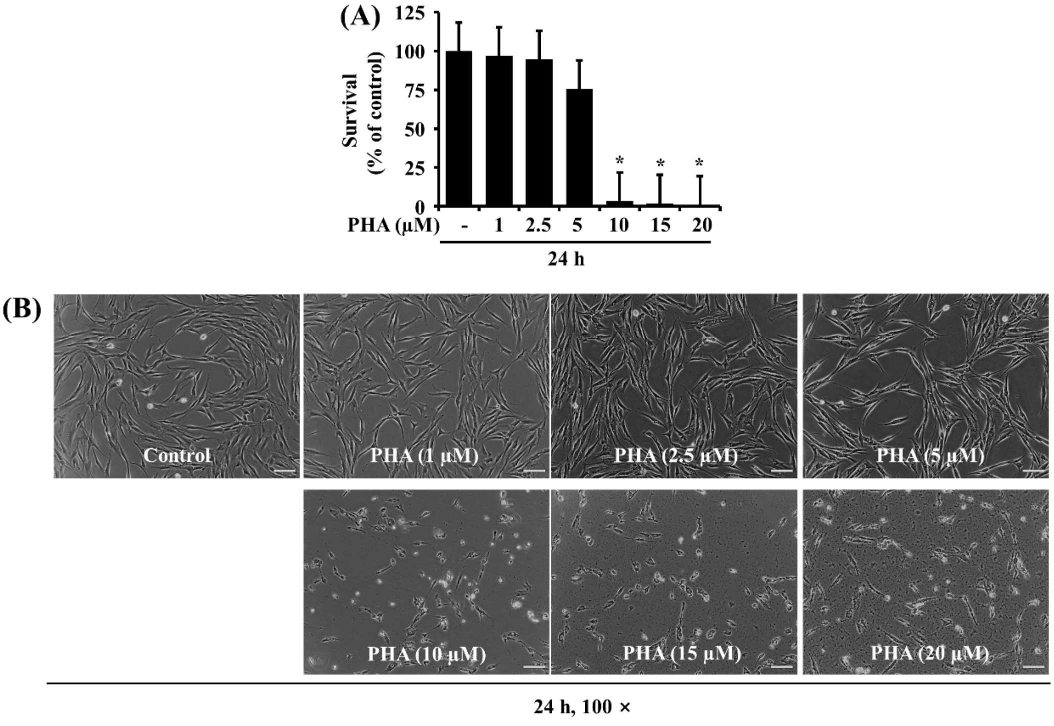

Figure S1. Effects of PHA on the growth of HGFs. (A) Normal human gingival fibroblasts (HGFs) were treated with vehicle control or PHA at the indicated concentrations at 24 h. The survival rate was determined by cell counting assay. Experiments were performed in triplicate. Data are the means ± SE of three independent experiments. * $p < 0.05$ compared to the value of vehicle control at the indicated time. (B) Images of the conditioned cells were obtained by phase-contrast microscopy, magnification, 100× (scale bar, 100 μm). Each image is representative of three independent experiments.
