# Supplementary material for: PHA-665752’s Antigrowth and Proapoptotic Effects on HSC-3 Human Oral Cancer Cells
Source: Int J Mol Sci. 2024 Mar 1;25(5):2871. doi: 10.3390/ijms25052871 (PMC10932316; doi:10.3390/ijms25052871)
Supplement: Supplementary file 1 [file ijms-25-02871-s001.zip › Table S1 Antibodies used in this study.pdf]

Table S1: List of antibodies used for Western blot analysis.

Antibodies	Dilution used	Source	Catalog no.
Primary antibodies			
p-cMET (Tyr1234/1235)	1:2,000	Cell signaling	3077
MET	1:2000	Cell signaling	3127
Mcl-1	1:2,000	Cell signalling	Sc819
Bcl-2	1:2,000	Santa Cruz Biotechnology	sc-509
p-Src (Y416)	1:2,000	Cell signaling	2101
Src	1:2,000	Cell signalling	2108
p-PKB (S473)	1:2,000	Cell signalling	9271
p-PKB (Thr308)	1:2,000	Abcam	9275
PKB	1:2,000	Cell signalling	9272
p-mTOR (S2448)	1:2,000	Cell signalling	2971
mTOR	1:2,000	Cell signaling	2983
p-EGFR (Tyr 1068)	1:2,000	Cell signaling	2234
EGFR	1:2,000	Cell signaling	2246
p-ERK1/2 (Thr202/Tyr204)	1:2,000	Cell signling	9101
ERK	1:2,000	Cell signaling	9102
HIF-1α	1:2,000	BD Biosciences	610958
HIF-1β	1:2,000	Santa Cruz Biotechnology	sc-17811
β-Actin	1:10,000	Sigma	A5441
Secondary antibodies			
Goat anti-rabbit IgG-HRP	1:5000	Jackson ImmunoResearch Laboratories	111-035-045
Goat anti-mouse-IgG-HRP	1:5000	Jackson ImmunoResearch Laboratories	115-035-062
